# P-1006. Oral vancomycin primary prophylaxis in transplant patients: universal administration vs. targeted screening for the prevention of C. difficile infection

**DOI:** 10.1093/ofid/ofaf695.1203

**Published:** 2026-01-11

**Authors:** Corinne Thompson, fainareti Zervou, Yanina Dubrovskaya, Dana Mazo, Michael Phillips, Sarah E Hochman

**Affiliations:** NYU Langone Health, New York, New York; new york university, New york city, New York; NYU Langone Health, New York, New York; New York University, New York, NY; New York University, New York, NY; NYU Langone Health, New York, New York

## Abstract

**Background:**

Asymptomatic colonization with *Clostridioides difficile* (CDiff) is common among hospitalized patients and may increase risk of symptomatic *C. difficile* infection (CDI) after hospitalization. Oral vancomycin prophylaxis (OVP) can reduce the risk of recurrent CDI and has been studied as primary prophylaxis in subsets of transplant recipients with no history of CDI. We compared CDI and vancomycin-resistant Enterococcus (VRE) rates among transplant recipients receiving primary OVP, CDiff screening and targeted OVP, and controls who received no CDiff screening or OVP.

**Figure:**
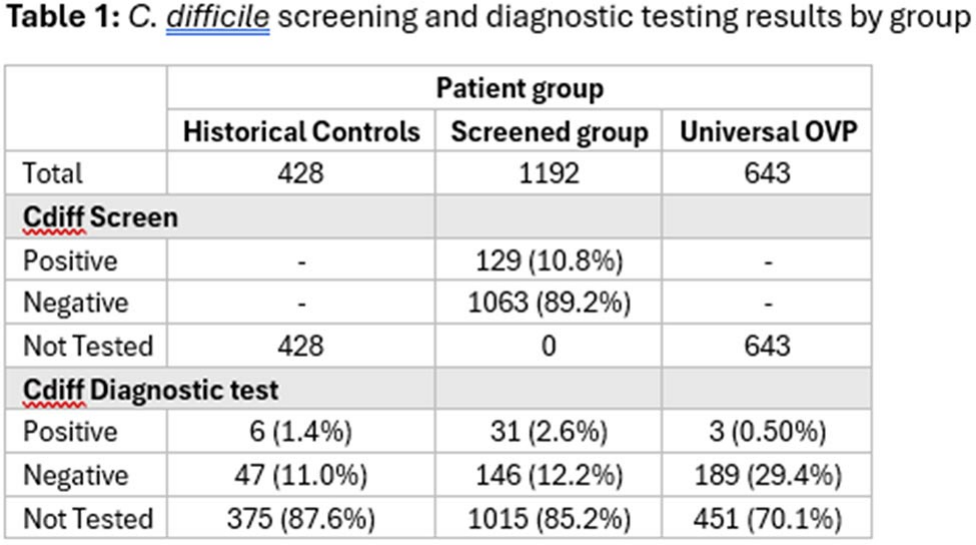
C. difficile screening and diagnostic testing results by group The proportion of each group who tested positive for CDiff screening or CDiff diagnostic testing.

**Figure:**
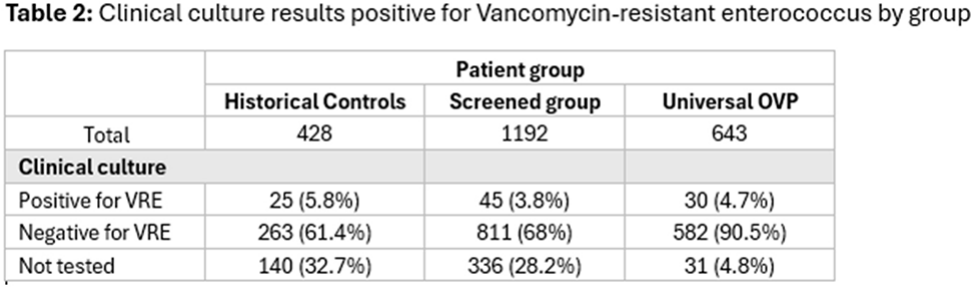
Clinical culture results positive for Vancomycin-resistant enterococcus by group The proportion of each group who had a clinical culture that was Vancomycin-resistant enterococcus

**Methods:**

Solid organ (SOT) and bone marrow transplant (BMT) patients were evaluated in three groups: (1) abdominal SOT patients screened for CDiff and given OVP if CDiff positive and receiving systemic antibiotics (Oct 2022 – Oct 2024), (2) BMT and thoracic SOT patients given universal OVP without screening (Oct 2022 – Oct 2024), and (3) historical control abdominal SOT patients without CDiff screening or OVP (Mar – Oct 2022). Patients were included from the date of first admission until date of final discharge from a transplant unit. CDiff screening was performed on admission. Hospital-onset CDI was monitored for the duration of the follow up and was defined as a positive CDiff stool test sent on hospital day 4 or later. Monitoring for clinical cultures positive for VRE occurred from the first admission to the transplant unit up to three months after the last admission. Rates of CDI and VRE were compared among groups by chi-square.

**Results:**

2263 patients were included; 1192 (53%) in the CDiff-screened group, 643 (28%) in the universal OVP group and 428 (19%) historical controls. Cdiff screening positivity was 10.8% in group 1, who also had higher rates of symptomatic CDI (2.6%, n=31/1192) compared with the universal OVP group (0.5%, 3/643) and historical controls (1.4%, 6/428) (p< 0.001). Rates of VRE were lower in patients screened for CDiff (3.8%, 45/1192) compared with those receiving universal OVP (4.7%, 30/643) and historical controls (5.8%, 25/428) (p< 0.001).

**Conclusion:**

Universal OVP effectively reduces the risk of CDI but is associated with higher rates of VRE in SOT and BMT patients.

**Disclosures:**

All Authors: No reported disclosures

